# Enhancing engagement between the population, environment, and climate research communities: the shared socio-economic pathway process

**DOI:** 10.1007/s11111-014-0202-7

**Published:** 2014-02-15

**Authors:** Lori M. Hunter, Brian C. O’Neill

**Affiliations:** Boulder, CO USA

**Keywords:** Climate change, Demography, Population, Scenarios, Shared socio-economic pathways, SSPs, IUSSP

## Abstract

Demographers have much to contribute to climate change science. This paper describes a new framework being developed by the climate research community that holds potential as an organizing tool for population–climate scholarship, as well as being useful for identifying demographic research gaps within the climate change field. The shared socio-economic pathways (SSPs) represent plausible alternative trends in the evolution of social and natural systems over the twenty-first century at the scale of the world and large regions. The SSPs can help identify population–environment research gaps by illuminating areas of intersection that will shape climate futures but require deeper scientific understanding—the association between urbanization and energy consumption is an example. Also, to vastly enhance the policy relevance of local case studies, the parameters outlined within the SSPs can offer a basic level of harmonization to facilitate generalization. In this way, the SSP framework can increase the relevance and accessibility of population research and, therefore, offer a mechanism through which demographic science can truly offer policy impact.

This paper briefly introduces a new scenario framework being developed by the climate research community that holds potential as an organizing tool for population–climate scholarship, as well as being useful for identifying demographic research gaps within the climate change field.

The framework is aimed at the development of integrated scenarios that bring together possible changes in future climate with changes in societal conditions in order to evaluate possible mitigation policies, adaptation policies, and impacts (Ebi et al. 2013; van Vuuren et al. 2013). The most relevant component of this framework for the population–environment community is a set of “shared socio-economic pathways” (SSPs) describing future societal conditions that are being collaboratively designed by a diverse social science community (O’Neill et al. 2013). In short, SSPs represent plausible alternative trends in the evolution of social and natural systems over the twenty-first century at the scale of the world and large regions. The pathways are being developed with the intent of improving prospects of harmonizing assumptions about future societal conditions across studies and, therefore, improving the generalizability and policy relevance of findings.

Demographers have important contributions to make with regard to development of the pathways, since understanding of demographic processes and the interaction between population–economy–environment is essential in the development of descriptions of plausible futures. In addition, demographers can use the pathways in their own research to facilitate comparison across studies and generalization of findings.

The following paper first describes the rationale for the SSPs and the process of pathway development. We then discuss the role of demographic research within SSP development, as well as the potential uses of the SSPs within demographic scholarship. We close with discussion of the SSPs in enhancing understanding of climate adaptation and mitigation challenges as related to different socio-economic futures.

## A new lens on socio-economic pathways toward different climate futures

In the past, a “linear process” has typified the generation of knowledge regarding the social dimensions of climate impact, adaptation, and vulnerability. In the first phase, integrated assessment modelers generated scenarios of emissions trends and drivers. These understandings of emissions then fed into climate projections, which have been in turn used by researchers interested in vulnerability, impacts, and potential adaptation strategies (Kriegler et al. 2012:812).

A different, “parallel” approach to development of scenarios has emerged in the past several years in which the generation of climate and societal futures are carried out at the same time, by separate research communities, and then integrated in a second step (Moss et al. 2010; van Vuuren et al. 2012; Kriegler et al. 2012). Key motivations are to shorten the process of developing and integrating alternative climate and societal futures, and to bridge and catalyze the “integrated assessment modeling” (IAM) and “impact, adaptation, and vulnerability” (IAV) research communities, allowing for scholarly understanding to emerge through an iterative process. In addition, the SSP framework is motivated by a desire to produce tangible outcomes of relevance to the ongoing assessment efforts of the IPCC.

**Fig. 1:**
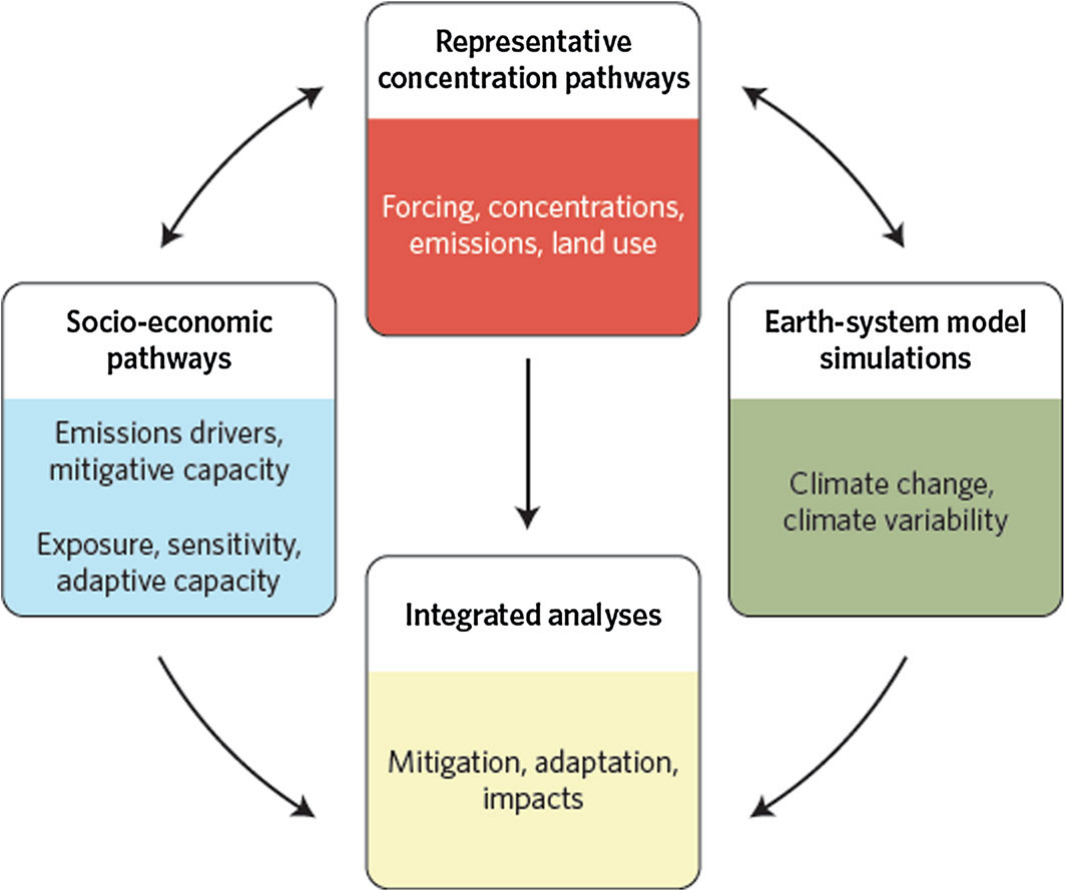
The parallel process conceptual diagram for the development of new, integrated scenarios of climate change. Van Vuuren et al. (2012) summarized the development of four new trajectories of radiative forcing over the twenty-first century, termed representative concentration pathways (RCPs). Future societal conditions and climate change simulations, consistent with these RCPs, will be integrated to investigate alternative mixes of climate change mitigation, adaptation, and impacts. From O’Neill and Schweizer 2011, figure adapted from Moss et al. (2010)

The new approach takes, as its start, scientific understanding of plausible futures of atmospheric composition—known as “representative concentration pathways” (RCPs; van Vuuren et al. 2012). Then, at the same time that the climate modeling community is producing simulations of climate change resulting from the RCPs (Taylor et al. 2011), a first set of SSPs has been developed covering a wide range of plausible socio-economic futures (see Fig. 1). This approach allows parallel development of climate science and the research aimed at understanding socio-economic determinants and implications.

The range of socio-economic factors important to include in these pathways is vast—demographic, economic, political, technological, and socio-cultural dimensions are all critical (see Table 1). In addition, conditions of ecosystems and ecosystem services that have been affected by human activity must also be considered, including air and water quality, biodiversity, and ecosystem form and function. Pathway development must therefore rely on current scientific understanding of the interaction of a range of socio-economic and biophysical factors. Indeed, given this complexity, a key challenge is the generation of a parsimonious set of socio-economic and ecological considerations within the SSPs.

**Table 1:** Illustrative factors considered in shared socio-economic pathways

Demographics
Population total and age structure
Urban versus rural populations, and urban forms
Other location information, such as coastal versus inland
Economic Development
Global and regional GDP, or trends in productivity
Regional, national, and sub-national distribution of GDP, including economic catch-up by developing countries
Sectoral structure of national economies. In particular, share of agriculture, and agricultural land productivity
Share of population in extreme poverty
Nature of international trade
Welfare
Human development
Educational attainment
Health, including access to public health and health care infrastructure
Environmental and Ecological Factors
Air, water, and soil quality
Ecosystem functioning
Resources
Fossil fuel resources and renewable energy potentials
Other key resources, such as phosphates and fresh water
Institutions and Governance
Existence, type, and effectiveness of national/regional/global institutions
Degree of participation
Rule of law
Technological Development
Type (e.g., slow, rapid, and transformational) and direction (e.g., environmental, efficiency, and productivity improving) of technological progress
Diffusion of innovation in particular sectors, e.g., energy supply, distribution and demand, industry, transport, and agriculture
Broader Societal Factors
Attitudes to environment/sustainability/equity and worldviews
Lifestyles (including diets)
Societal tension and conflict levels

*Source* O’Neill et al. 2013

The shared socio-economic pathways include both quantitative and qualitative elements. Values and trends for a core set of variables—prominently including demographic variables—yield quantitative profiles and projections for the pathways. Qualitative narratives describe storylines, allowing consideration of a wider array of socio-economic factors and interactions. At present, five SSPs have emerged as core representative pathways (see Table 2), with extended SSPs offering varieties within these broad futures.

**Table 2:** Five core shared socio-economic pathways

*SSP1 Sustainability*
The world shifts gradually, but dramatically, toward a more sustainable path. Management of the global commons slowly improves, educational and health investments accelerate the demographic transition, and concerns with economic growth shift toward the implications for human well-being. Overall energy and resource use is reduced over the longer term, and renewables become more attractive.
*SSP 2 Middle of the Road*
Development and income growth proceeds unevenly, with only some countries making relatively good progress. Global and national institutions work toward but make slow progress in achieving sustainable development goals. Technological developments proceed apace, but without fundamental breakthroughs. Global population growth is moderate; education investments are not high enough to accelerate the transition to low fertility rates in low-income countries.
*SSP 3 Fragmentation*
Growing interest in regional identity and concerns about competitiveness and security push countries to increasingly focus on domestic or, at most, regional issues. Policies are oriented toward security, and countries focus on achieving energy and food security goals within their own region, at the expense of broader-based development. Population growth is low in industrialized and high in developing countries. Inequities are high, especially in developing countries. There is growing resource intensity and fossil fuel dependency along with difficulty in achieving international cooperation.
*SSP 4 Inequality*
In this world, inequalities increase, both between and within countries. Vulnerable groups are largely outside the mainstream globalized economic system and have little representation in national and global institutions, which emphasize international competitiveness. This is a world with low social cohesion, and regularly in social conflict and unrest. Power becomes more concentrated in a relatively small political and business elite, even in democratic societies. Energy companies diversify their energy sources to hedge against price fluctuations, investing in carbon-intensive fuels such as coal and unconventional oil, but also low-carbon energy sources.
*SSP 5 Conventional Development*
Driven by the economic success of industrialized and emerging economies, this world places increasing faith in competitive markets, innovation, and participatory societies to produce rapid technological progress and development of human capital as the path to sustainable development. Global markets are increasingly integrated, and there are strong investments in health, education, and institutions to enhance human and social capital. At the same time, the push for economic and social development is coupled with the exploitation of abundant fossil fuel resources and the adoption of resource and energy intensive lifestyle around the world.

*Source* Based on initial drafts of narratives in O’Neill et al. 2012a, to be updated in O’Neill et al. (under review)

As an example, imagine a SSP characterized by high levels of economic growth and improved human capital, combining to yield overall lower population growth. The pathway could also be characterized by high energy demand met predominantly with carbon-based fuels. In the absence of climate policy, such a scenario would lead to a far different climate future than one characterized by rapid technological change directed toward environmentally friendly processes (Kriegler et al. 2012; O’Neill et al. 2012a).

Several methodologies have been applied to identify central SSP elements and narratives, including expert elicitation (Schweizer and O’Neill 2013), creation of large numbers of candidate pathways (Schweizer and O’Neill 2013; Rozenberg et al. 2013), and group consensus processes (O’Neill et al. 2013). Although operating at the global and regional scales, the SSPs make use of scientific understanding of socio-ecological interactions at finer resolution, including national, subnational, state, and community-level scholarship. Thanks to input from demographic researchers throughout this development process, the SSPs include informed population, education, and urbanization projections at the national level with global coverage. Global, spatially explicit population projections are also currently being developed.

An example of demographic research incorporated within the SSPs is provided by KC and Lutz (see their contribution to this special issue; see Lutz 2013, for a summary). The authors translate the SSP narratives into five alternative demographic scenarios providing projections by age, sex, and level of education for 171 countries up to 2100. In addition, Jiang and O’Neill (under review) translate the SSP narratives into alternative projections of national-level urbanization. The new demographic scenarios, which are available online at https://secure.iiasa.ac.at/web-apps/ene/SspDb along with other quantitative elements of the SSPs and a discussion of assumptions and methodology, present a major step forward as compared to the earlier SRES scenarios that only considered total population size (Nakicenovic et al. 2000).

The SSPs can be applied in research studies and integrated scenario development, allowing for harmonization of key inputs. Given a sufficient number of studies using common assumptions about future climate and societal conditions, broad conclusions about options for responding to climate change will be able to be drawn in a way that is supported by a diverse research base. In this way, SSPs will provide a common framework from which different research communities can engage (Kriegler et al. 2012; O’Neill et al. 2013).

## Demographic research and development of shared socio-economic pathways

As noted, population–climate researchers, and demographers more generally, have already contributed projections, scholarship, and expert comment toward SSP development. Indeed, a vast array of demographic research examines the included socio-economic processes, and their interactions, even if not explicitly engaging climate.

Still, a key challenge for the demographic research community is to determine whether there are demographic futures not well represented in the current set of SSPs. Are there additional demographic scenarios that should be considered in the SSPs—perhaps a wider range of outcomes? Different combinations of trends? Surprises?

Also, the demographic dynamics assumed in SSPs obviously do not act in isolation. Demographic research can help ensure that the demographic assumptions are consistent with other scenario elements (see Jiang’s paper in this special issue). As an example, SSP storylines include assumptions regarding urbanization and fertility—a connection with substantial research coverage (e.g., Shapiro and Tambashe 2002; White et al. 2008).

As another example, a substantial amount of research links urbanization to economic development and GDP, yet few of these interactions have been incorporated in integrated assessment models (Krey et al. 2012). Even so, new efforts have been made to explain the “no growth” urbanization experienced in sub-Saharan Africa throughout the 1980s and 1990s. Fox (2012) argues, for example, that technology and institutional innovations represent key determinants of urbanization through resulting health gains and enhanced food security, especially in urban regions. Such de-coupling of urbanization from GDP and economic development, particularly in some global regions, has important implications for global emission models that consider such interactions. These nuanced discussions of urbanization determinants also deserve a place in the narratives describing SSPs.

Demographic research also reveals that both urbanization and aging are linked to energy use patterns, a key determinant of future emissions (O’Neill et al. 2012b). In industrialized settings, aging may reduce long-term emissions by up to 20 percent through decreased economic productivity and reduced consumption. Urbanization in less developed settings, however, may counteract these reductions by yielding a 25 percent increase in emissions due to the heightened consumption and economic productivity associated with urban living (O’Neill et al. 2010). The demographic perspective and toolkit have also shed light on household and living arrangements and their potential future changes (Zeng et al. 2013). Since households are primary units of consumption and consumption drives emissions, understanding these demographic shifts is also important for SSP development.

Population researchers are also making important advances in the measurement and spatial projection of urbanization and urban populations. As examples, the Global Rural Urban Mapping Project (GRUMP) represents the first spatial rendering of global urban areas with population estimates, making use of satellite data. In addition, researchers are generating new methods for estimating and forecasting urban and city population that combine demographic and econometric techniques and use survey, census, and spatial data (Montgomery and Balk 2011). Understanding climate vulnerability along China’s coast provides an example of these endeavors’ importance. While China’s population growth between 1990 and 2000 was 1.04 %, urban growth was double at 2.33 %, with particularly high concentrations in urban coastal regions (Smith 2011). Such spatial precision in urban estimates and projections can usefully be engaged in development of SSPs.

## Using the shared socio-economic pathways in population–environment research

The SSPs can be used as a framework by the population–environment research community for identifying research areas that could usefully contribute to this important effort. As an example, we could ask: Which demographic factors and relationships can be reliably projected quantitatively and can we do better than we are doing now? The ongoing efforts to spatially represent future urban populations represent such a contribution. The demographic research community can also provide insight into whether the SSPs neglect important aspects of regional or global demographic futures and/or population–climate interactions.

Also, to vastly enhance the policy relevance of local studies, the SSPs can offer a basic level of harmonization that will facilitate generalization across a range of case studies. Specifically, the SSPs can be used for local analyses by providing guidance on global patterns to be linked to context-specific case studies. The intent is not that the SSPs offer deterministic parameters but rather assumptions that can frame the variation examined within local settings—and, in this way, provide essential insight into the implications of different pathways.

More specifically, demographers working in particular local settings can contribute to understanding the implication of climate futures by framing their research, at least in part, with SSP storylines. Indeed, the many facets of the SSP storylines offer unlimited research questions for demographers—and answers to the questions would aid in refinement of the pathways and understanding of related mitigation and adaptation challenges.

One of the authors (LH) can reach to her own collaborative research in rural South Africa as an example. This work has been examining migration as a livelihood strategy among natural resource-dependent rural households at the Agincourt Health and Demographic Surveillance Site (Hunter et al. 2013; Leyk et al. 2012). A useful extension would be to consider how the patterns that have been identified might shift under different future socio-economic pathways. As others studying migration–environment connections do similarly, this research can more usefully be linked to generalize with regard to future climate challenges under different scenarios. And more broadly, by doing so, we can better understand how the patterns described in broad SSPs might vary across specific local areas characterized by different development level, economic contexts, or other socio-cultural distinctions.

Importantly, such context-specific research can also help in refinement of the pathways themselves. The SSPs are intended to evolve and to be refined through iterations with researchers and their research findings. Therefore, the broad pathways and context-specific research are reciprocally related. The SSPs can provide a useful harmonization framework for local research, while context-specific research will also usefully inform broadscale scenario (re)development.

And finally, while the initial core set of SSPs has been identified, researchers are encouraged to develop variants of these five SSPs—including extensions to additional variables and/or local regions. Importantly, research studies need not examine the entire socio-climate system to contribute to this process. Instead, given relatively harmonized objectives and boundary parameters, research focused on portions of the socio-ecological systems that shape climate futures can become integrated into holistic modeling efforts that feed more directly into policy. An example is the Agricultural Model Intercomparison Project (AgMIP) that is extending SSPs to make them more specific for agricultural studies. In the end, these different research applications should inform future iterations of the SSPs themselves.^1^

## The SSPs as a means of identifying challenges to climate mitigation and adaptation

Another central motivation in generating SSPs is further understanding of challenges to climate change mitigation and adaptation. Indeed, the initial aim in crafting a core set of SSPs was to span a wide range of outcomes in mitigation and adaptation challenges.

**Fig. 2:**
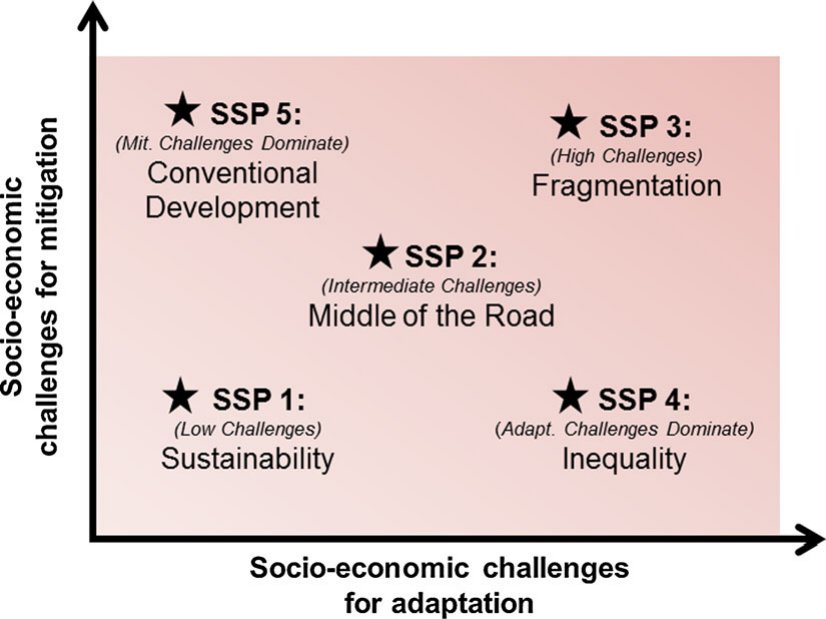
The “challenges space” spanned by SSPs (based on O’Neill et al. 2012a, Fig. 1), divided into five “domains” with one SSP located within each domain, represented by a star

Mitigation represents “technological change and changes in activities that reduce resource inputs and emissions per unit of output”. (IPCC 2011:962) Enhanced use of renewable energy is, for example, a mitigation option if greenhouse gas emissions are reduced as compared to other forms of energy production. Adaptation is defined, in human systems, as “the process of adjustment to actual or expected climate and its effects, in order to moderate harm or exploit beneficial opportunities”. (IPCC 2012:5) Using mitigation and adaptation challenges as axes of interest for development of the SSPs, a “challenges space” can be envisioned as represented in Fig. 2.

We could also think of the initial SSPs as hypotheses asking about the relative importance of different processes in shaping mitigation and adaptation challenges. Research can test these hypotheses. As an example, would a highly urbanized world be better or less able to adapt to or mitigate future climate change?

Other useful and interesting demographic scholarship would contrast variables with regard to their contributions to mitigation and adaptation challenges. Which demographic factors contribute most to the future challenges—what is the relative importance of migration, aging, progress in educational attainment? How does this vary regionally or by development level? What can existing case study literature tell us about these associations and challenges already?

As illustration, consider the brief SSP narratives presented above. One illustrative pathway described a future characterized by high levels of economic growth, improved human capital, lower population growth, and reliance on carbon-based fuels. In Fig. 1, this may represent SSP5, “conventional development”, a world reasonably well equipped to adapt due to higher levels of human capital yet facing substantial mitigation challenges due to emission-intensive energy dependence. On the other hand, an illustrative narrative for SSP4, “inequality”, could represent emphasis on low carbon energy technologies in key emitting regions, thereby facilitating mitigation. Yet, global inequality could be high with some economies relatively isolated, lessening adaptive capacity. These examples and additional narratives are usefully described by Kriegler et al. (2012:817).

The logic here is that there is substantial utility in characterizations of socio-economic pathways that would make mitigation and adaptation relatively hard or relatively easy. And linking these pathways to these policy-relevant domains enhances the usefulness of the entire research endeavor.

## Conclusion

A variety of research communities, including demographers, have come together to generate the SSPs, plausible alternative trends in the evolution of social and natural systems over the twenty-first century at the scale of the world and large regions. Population researchers must continue to engage in future iterations and extensions of the SSPs since demographic patterns and processes certainly play fundamental roles in determining the planet’s climate future and in shaping challenges related to climate change mitigation and adaptation.

In addition, demographers are well positioned to make use of the SSPs in our own scholarship and thereby offer important contributions to understanding the drivers and implications of various climate futures. Using the SSP framework will enhance the relevance and accessibility of population scholarship to climate scientists and policymakers. In this way, given the enormity of the climate challenge, the SSPs offer a window of opportunity for population research to truly make a difference.

## Footnotes

1 Ideas for where and how to extend the SSPs are very welcome and can be communicated to an ad hoc committee that has been set up to facilitate the scenario process—the International Committee On New Integrated Climate change assessment Scenarios (ICONICS), see https://www.isp.ucar.edu/iconics.
